# A Radial Basis Function Neural Network Approach to Predict Preschool Teachers’ Technology Acceptance Behavior

**DOI:** 10.3389/fpsyg.2022.880753

**Published:** 2022-06-09

**Authors:** Dana Rad, Gilbert C. Magulod, Evelina Balas, Alina Roman, Anca Egerau, Roxana Maier, Sonia Ignat, Tiberiu Dughi, Valentina Balas, Edgar Demeter, Gavril Rad, Roxana Chis

**Affiliations:** ^1^Faculty of Educational Sciences, Psychology and Social Sciences, Center of Research Development and Innovation in Psychology, Aurel Vlaicu University of Arad, Arad, Romania; ^2^College of Teacher Education, Cagayan State University, Tuguegarao, Philippines; ^3^Faculty of Engineering, Aurel Vlaicu University of Arad, Arad, Romania

**Keywords:** technology acceptance model, preschool education, behavioral modeling, neural networks, radial basis function

## Abstract

With the continual development of artificial intelligence and smart computing in recent years, quantitative approaches have become increasingly popular as an efficient modeling tool as they do not necessitate complicated mathematical models. Many nations have taken steps, such as transitioning to online schooling, to decrease the harm caused by coronaviruses. Inspired by the demand for technology in early education, the present research uses a radial basis function (RBF) neural network (NN) modeling technique to predict preschool instructors’ technology usage in classes based on recognized determinant characteristics of technology acceptance. In this regard, this study utilized the RBFNN approach to predict preschool teachers’ technology acceptance behavior, based on the theory of planned behavior, which states that behavioral achievement, in our case the actual technology use in class, depends on motivation, intention and ability, and behavioral control. Thus, this research design is based on an adapted version of the technology acceptance model (TAM) with eight dimensions: D1. Perceived usefulness, D2. Perceived ease of use, D3. Perceived enjoyment, D4. Intention to use, D5. Actual use, D6. Compatibility, D7. Attitude, and D8. Self-efficacy. According to the TAM, actual usage is significantly predicted by the other seven dimensions used in this research. Instead of using the classical multiple linear regression statistical processing of data, we opted for a NN based on the RBF approach to predict the actual usage behavior. This study included 182 preschool teachers who were randomly chosen from a project-based national preschool teacher training program and who responded to our online questionnaire. After designing the RBF function with the actual usage as an output variable and the other seven dimensions as input variables, in the model summary, we obtained in the training sample a sum of squares error of 37.5 and a percent of incorrect predictions of 43.3%. In the testing sample, we obtained a sum of squares error of 14.88 and a percent of incorrect predictions of 37%. Thus, we can conclude that 63% of the classified data are correctly assigned to the models’ dependent variable, i.e., actual technology use, which is a significant rate of correct predictions in the testing sample. This high significant percentage of correct classification represents an important result, mainly because this is the first study to apply RBFNN’s prediction on psychological data, opening up a new interdisciplinary field of research.

## Introduction

Across the world, the COVID-19 epidemic has forced the closure of schools and preschools in 20 nations and 19 countries. This impacted 49.8 million children, ranging from toddlers to high school students, who had an immensely interrupted past school year, culminating in school closures (Hoofman and Secord, 2021; Jehi et al., 2022). The epidemic has had a significant impact on schooling in the region, as well as worsened socioeconomic inequality (Deaconu and Olah, 2022).

The preschool education environment is not just the place for academic instruction but also for developing socio-emotional abilities, interaction with peers, and receiving social assistance. School closures have affected not only the educational process but also access to other extracurricular activities, school meals, welfare help, and referral to essential medical and psycho-social services. Preschool teachers, school administrators, education authorities, and local and national decision-makers faced a huge problem during the COVID-19 lockdown (Cantiani et al., 2021; Charernnit et al., 2021; Khamidullina et al., 2021; Teng et al., 2021; Ullmann et al., 2021). If the problem is not tackled, the consequences for children, young people, families, communities, and society at large will be felt for the rest of their lives, both socially and economically. As a result, strengthening the educational system’s resilience by planning a high-quality inclusive education based on technology usage in class should be a top priority in the coming months and years. A recent review of the literature (Zomer and Kay, 2016) on the use of technology in early childhood education revealed four key content areas of focus, namely, literacy, engagement, social interactions, and mathematics. Targeting the impact of technology on literacy skills and the use of technology in class revealed statistically significant gains in phonological awareness, vocabulary, general literacy, reading compression, and the concept of print; a positive relationship between the use of technology and engagement; and a positive impact on social interactions like cooperating, sharing, and collaborating. Also, technology helped to improve mathematical skills such as numeracy skills, counting, and identifying shapes. The average effect size was 0.71, indicating that the impact of technology on younger children was meaningful (Zomer and Kay, 2016).

Inspired by the demand of technology for early education, the present research uses a radial basis function (RBF) neural network (NN) modeling technique to predict preschool instructors’ technology usage in class based on recognized determinant characteristics of technology acceptance. In this regard, this study utilized the RBFNN approach to predict preschool teachers’ technology acceptance behavior, based on the theory of planned behavior, which states that behavioral achievement, the actual technology use in class in our case, depends on motivation, intention and ability, and behavioral control; thus, this research design is based on an adapted version of technology acceptance model (TAM) with eight dimensions: D1. Perceived usefulness, D2. Perceived ease of use, D3. Perceived enjoyment, D4. Intention to use, D5. Actual use, D6. Compatibility, D7. Attitude, and D8. Self-efficacy. According to the TAM, actual usage is significantly predicted by the seven other dimensions used in this research. Instead of using the classical multiple linear regression statistical processing of data, we opted for a NN based on RBF approach to predict the actual usage behavior. The use of RBF networks is distinguished from other NNs due to their universal approximation and faster learning speed. An RBF network is a type of feed-forward NN composed of three layers, namely the input layer, the hidden layer, and the output layer. As such, this RBFNN will provide empirical evidence on the speed of learning process using technology in the preschool learning environment.

## Digital Instruments in Preschool Education

Given the enormous impact of modern technology tools on the education system, numerous teaching staff are becoming more willing to incorporate them into their teaching activities (Blackwell et al., 2014; Bourbour Hosseinbeigi, 2020; Undheim, 2021). It is necessary to surround the child with such an environment and such a system of relationships that stimulates the most diverse independent activities of the child and to form exactly what the child at the right time is able to perform (Plumb and Kautz, 2016; Yilmaz, 2016; Su and Yang, 2022).

Modern educational technologies, as never before, are based on the intellectual development of children, and learning through play is fully in line with this concept (Luo et al., 2021). Modern requirements for preschool education guide teachers toward the development of education and dictate the need to use new technologies that synthesize elements of cognitive interaction, play, search, and educational development of preschoolers, mastering them in constructive ways and means of interaction with the people around them in accordance with the tasks set by the modern educational standards (Wang et al., 2010; Salomon, 2012; Baccaglini-Frank and Maracci, 2015; Sullivan and Bers, 2016; Rau, 2017, 2020; Behnamnia et al., 2020).

Studies claimed that digital tools improved teaching and learning in the preschool, basic, and higher education levels (Magulod, 2018, 2019, Fan and Tan, 2019; Otterborn et al., 2019; Xie et al., 2019; Tarihoran et al., 2022). Thus, interactive learning with the help of technology is, without a doubt, an interesting, creative, and promising direction of pedagogy. It helps to realize all the possibilities of preschool children, taking into account their psychological abilities. The use of interactive technology makes it possible to enrich children’s knowledge and ideas about the world around them and about relationships with peers and adults and encourages children to actively interact in the system of social relationships and develop key competences (Aydogdu and Kelpšiene, 2021; Hong et al., 2021).

Forth key competences are a versatile, transferable set of knowledge, abilities, and attitudes that all people require for personal fulfillment and growth, social inclusion, and obtaining work. They must have emerged at the completion of compulsory school and serve as a basis for learning as part of lifetime learning. This study is a first step toward educators’ and instructors’ usage of technology. The usage of e-technologies in the preschool learning environment saves time and energy most of the time, effortlessly capturing the interest of toddlers. Teachers must adhere to their pedagogical beliefs while selecting digital technologies (the degree of interaction, communication and collaboration, the development of 21st century skills, etc.). Preschoolers will benefit from a more comfortable learning environment, but this will necessitate the assistance of adults (parents, in the case of home learning). The exchange of best practices and help from peers is a motivating factor for pre-school teachers to become effective and efficient in their teaching practices considering that their role is very critical in the foundational years of children.

Although research examining technology acceptance has generally focused on students and teachers, we are unaware of any empirical studies to determine the preschool teachers’ technology acceptance intention and the influential factors, especially in the context of a global public health emergency, except the research by Hong et al. (2021) on preschool teachers’ technology acceptance during the COVID-19 period, in Chinese context. This identified empirical research gap motivated us to examine Romanian preschool teachers’ intentions toward computer-based educational technology and the determinants in Romanian early childhood education system.

Romanian society is constantly developing, with huge changes in the educational system, particularly in preschool education. The context of the epidemic compelled these adjustments, albeit forcefully. To bring consistency to the teaching act in this dynamic setting, each instructor can pick from a variety of modern digital tools depending on their preferences and personal expertise. Although online education cannot completely replace the traditional education, it can serve as a useful supplement. The goal of integrating new technologies into education is to improve the educational system’s performance by favorably influencing the outcomes of the instructional–educational process.

In this regard, on the individual level, the theory of planned behavior is the most robust psychological theory that links beliefs to behavior, in the context of our research linking beliefs about integrating technology in Early Childhood Education and Care (ECEC) to actually implementing activities assisted by technology with children in kindergartens. In order to enhance the technology acceptance and use in ECEC, according to the theory of planned behavior, one can instill positive beliefs and attitudes about technology use like perceived usefulness, perceived ease of use, perceived enjoyment, compatibility, positive attitude, and self-efficacy.

If past research focused at this predictive model of technology acceptance by preschool teachers in a linear fashion (Hong et al., 2021), our research focuses on a predictive model that deals with dynamic relationships between psychological determinants: perceived usefulness, perceived ease of use, perceived enjoyment, compatibility, positive attitude, and self-efficacy, according to the TAM.

Specifically, this research is focused on the analysis of whether RBF neuronal network modeling approach is a robust prediction technique with actual use by preschool teachers based on the dynamic relationships between psychological determinants, namely perceived usefulness, perceived ease of use, perceived enjoyment, compatibility, positive attitude and self-efficacy.

The following section will describe the TAM and main psychological components that this research has further taken into consideration.

## Technology Acceptance Model Development and Adaptation in Preschool Education

The use of the TAM has been developed by Davis (1989), which is a modification of the theory of reasoned action (TRA). The model was designed to explain how individuals use new technology by creating a causal relationship among their beliefs, perceptions, attitudes, intentions, and behaviors. The two major constructs in TAM as defined by Davis (1989) are perceived utility, which is the degree to which individuals believe that using a particular system leads to higher performance, and perceived ease of use, which is the degree to which individuals expect the use of a particular system to be effortless. The behavioral intention of use determines the usage of a system, which is influenced by the concurrent action of two factors: perceived usefulness and attitude. Personal ideas about perceived utility and simplicity of use influence attitudes toward use.

The direct relationship between perceived utility and intent shows that people form their behavioral intentions for use based on a cognitive appreciation of how using the system will help in improving their performance. Perceived utility is influenced by perceived ease of use because if all other influences are equal, then the easier the system is to use, the more useful the system is. Thus, if a system that is considered useful by the individual is difficult to use, then the possible performance that can be obtained through it is blurred by the effort that must be made to use it. External variables (system characteristics, user characteristics, nature of the development process, etc.) define the link between, on the one hand, the beliefs, attitudes and intentions of the TAM and, on the other hand, individual differences, constraints, context-specific use, and managerially controllable interventions that influence the system use (Davis et al., 1989; Davis, 1993; Venkatesh and Davis, 2000). Although similar to TRA, the TAM differs from TRA in several theoretical aspects (Davis et al., 1989). In the case of TRA, the behavioral intention is determined by the subjective norm and attitude, while in the case of the TAM, this is determined by the perceived utility and attitude. In the experiments performed by Davis et al. (1989), the subjective norm construct in TRA was not statistically significant, so it was excluded from the TAM. In the TAM, the perceived utility constructs and the perceived ease of use are fundamental and distinct, but they are correlated constructs. Perceived utility is the determining factor of intent to use, and perceived ease of use is a significant secondary determinant. The use of a system can be reasonably predicted from its behavioral intentions.

Numerous studies have identified the need to include new variables and expand the model with new constructs in order to understand how people adopt new technologies. Davis et al. (1992) used the motivational theory and proposed a new model called the “motivational model” (MM). The MM is based on two key concepts: extrinsic motivation and intrinsic motivation. Both MM and TAM each include a construct that emphasizes the individual’s gain in relation to the use of technology, respectively, extrinsic motivation, in the case of MM, and perceived utility, in the case of the TAM. In the first testing of the models (Davis et al., 1989, 1992), the constructs were measured using variables with the same name and then included under the umbrella of the same concept (Davis et al., 1992). The original TAM model does not include intrinsic motivation. Venkatesh (2000) developed and tested an integrated model by including the concept of intrinsic motivation in the TAM.

Van der Heijden (2004) included the construct “perceived enjoyment,” highlighting the positive link between ease of use and pleasure (enjoyment), as well as between pleasure and intention to use (Chesney, 2006). In the original TAM model (Davis et al., 1989), the attitude construct was validated as a partial mediator between personal beliefs (perceived usefulness and perceived ease of use) and system use. In subsequent studies, the attitude construct was eliminated on the basis of practical arguments (Venkatesh, 2000; Venkatesh and Davis, 2000; Venkatesh et al., 2003). As a result, most researchers have proposed direct links from perceived ease of use and perceived utility to intended use, respectively, without including attitude as a mediating factor (Venkatesh et al., 2003; Van der Heijden, 2004). As Dillon and Morris (1998) have shown, the TAM provides useful feedback on whether or not the users will accept a new technology.

The last two dimensions introduced in the TAM are compatibility and self-efficacy.

The TAM that has been used by numerous studies to understand the technological acceptability will help forecast how new information resources will be used (Numondjonovna, 2021; Kartal et al., 2022; Mohebi, 2022; Zhou, 2022). Confidence in the use of technology, according to the study, can lead to enhanced personal control, flexibility, and competent information utilization. As a result, greater knowledge can lead to increased production. The TAM has been criticized for a number of reasons, but it is a useful overall framework that has been found to be compatible with a number of studies looking at the factors that influence people’s readiness to embrace new technology. The use of the TAM scale in this study will provide empirical evidence on the technological literacy of preschool teachers in the Romanian context. Numerous studies which have used the TAM to understand the technical acceptability will help forecast how new information resources will be used (Numondjonovna, 2021; Kartal et al., 2022; Mohebi, 2022; Zhou, 2022).

While admitting the relevance of individual ideas about a technology’s compatibility, technological acceptance of a theoretical and empirical study has generated mixed outcomes. The research by Karahanna et al. (2006) aimed to advance the conceptualization of this crucial belief in technological acceptance. Authors provide a detailed compatibility definition focused on four distinct and separable constructs: preferred work style, existing work practices, prior experience, and with values, thus establishing a framework of compatibility beliefs as well as operational measurements and placed them in the TAM. According to this framework, the higher the teachers’ compatibility with using technology in the class, the higher the teachers’ adoption of technology.

Bandura (1977) defines self-efficacy as a person’s conviction or judgment in their ability to organize and execute necessary actions for achieving a specific performance. As a result, teacher’s self-efficacy is an educator’s ultimate conviction in his or her capacity to do various educational responsibilities (Dellinger et al., 2008). Teachers with high self-efficacy views are more likely to embrace ideas and execute techniques to enhance their methods through the use of technology, according to Allinder (1994).

During the COVID-19 epidemic, online education and implicitly technology assisted education have become major tools in the school system (Iivari et al., 2020). Many nations have made steps to decrease the harm caused by coronaviruses, such as transitioning to online schooling (Jandrić, 2020; Zhang et al., 2020). To prevent the spread of COVID-19, all preschools in Romania, like many other nations across the world, were obliged to migrate to a hybrid version of e-learning. As a result, all preschool instructors were forced to rapidly and successfully acquire and implement instructional strategies with which they were unfamiliar (Hodges et al., 2020; Rapanta et al., 2020).

Teachers’ willingness and acceptance of technology are critical factors in the effective implementation of technology into the teaching process (Yuen and Ma, 2008). To guarantee the smooth growth of online preschool education throughout the pandemic, it is critical to investigate preschool instructors’ readiness to adopt technology as well as the elements that influence it. However, researchers have not devoted much attention to this subject thus far. As a result, there is a rising need to investigate and study their behavior intentions toward adopting technology. Our results would give insights into preschool instructors’ adoption of and usage of technology throughout the pandemic, which would aid in preschool teaching consistency.

The TAM validity in an educational environment was demonstrated in studies done on a sample of pre-service and gymnasium teachers (Pynoo et al., 2011; Teo and Noyes, 2014). Thus, the preceding study is primarily focused on different educational sectors (Chang et al., 2017; Granić and Marangunić, 2019; Yang and Wang, 2019), with little studies on preschool education.

Although several earlier research have explored and characterized determinants of behavioral intention to use technology, little is known about the antecedent factors of education technology acceptability for preschool instructors, except for the study of Hong et al. (2021). Building and testing a model with numerous impacting elements leads to a more thorough knowledge of the preschool teacher’s technology acceptance. This research used the modified TAM to model preschool teachers’ behavioral adoption of technology, which was motivated by earlier literature.

Thus, the TAM suggested by us will further take into consideration the following dimensions: perceived usefulness, perceived ease of use, perceived enjoyment, intention to use, actual use, compatibility, attitude, and self-efficacy.

## The Present Study

Following a review of the literature, we observed that the TAM is broadly applicable, giving a solid theoretical framework as well as an empirical support for our study. The use of NN algorithms for forecasting real technology use based on recognized psychological variables has never been published in a study.

Inspired by the demand of technology into early education, the present research uses a RBFNN modeling technique to predict preschool instructors’ real technology usage in class based on recognized determinant characteristics toward educational technology.

Data-driven techniques have grown increasingly popular as a successful modeling tool in recent years due to the continued development of artificial intelligence and sophisticated algorithms. Because of its simple structure and outstanding nonlinear function approximation capacity, the RBFNN is often used, especially in data classification and nonlinear system modeling (Yang et al., 2013; Han et al., 2015; Xiong et al., 2015; Xu et al., 2016). When the nature of relationship between research variables is complex, nonlinear, and difficult to specify in structural equations, NNs may be utilized as statistical tools for data processing and may even outperform standard statistical processes in classification and completion tasks (Garson, 1998; Levine, 1998; Gafarov et al., 2021).

In this regard, on the individual level, the theory of planned behavior is the most robust psychological theory that links beliefs to behavior, in the context of our research linking beliefs about integrating technology in ECEC to actually implementing activities assisted by technology with children in kindergartens. In order to enhance the technology acceptance and use in ECEC, according to the theory of planned behavior, one can instill positive beliefs and attitudes about technology use like perceived usefulness, perceived ease of use, perceived enjoyment, compatibility, positive attitude, and self-efficacy.

This research design is based on an adapted version of TAM with eight dimensions: D1. Perceived usefulness, D2. Perceived ease of use, D3. Perceived enjoyment, D4. Intention to use, D5. Actual use, D6. Compatibility, D7. Attitude, and D8. Self-efficacy. According to the TAM, actual usage is significantly predicted by the seven other dimensions used in this research. Instead of using the classical multiple linear regression statistical processing of data, we have opted for a RBFNN approach to predict the actual usage behavior in terms of correct classification percentage.

Specifically, this research is focused on the following research question:

Is RBF neuronal network modeling approach a robust prediction technique for determining the actual usage of technology by preschool teachers based on the dynamic relationships between the psychological determinants: perceived usefulness, perceived ease of use, perceived enjoyment, compatibility, positive attitude, and self-efficacy.

## Materials and Methods

### Participants

This study included 182 preschool teachers who were chosen at random from a project-based national preschool teacher training program called Inclusive and Qualitative Early Education, which began in Romania in the spring of 2021 during the COVID-19 pandemic. The Romanian Ministry of Education launched a countrywide preschool instructor training program for 2,000 preschool teachers. A total of 182 preschool teachers were engaged in online education and used educational technology in their classrooms during the COVID-19. The participants were selected from the official list of preschool teachers requested from the Romanian Ministry of Education. The 182 teachers were screened from the bottom for those who possess the following professional characteristics: (1) must have been teaching preschool for 5 years since they already possessed the pedagogical experience in dealing with children; (2) must have attended trainings on educational technology in the national levels; (3) must have conducted demonstration teaching with the integration of technology in the classroom during in-service training programs; and (4) must have been active in doing research. Using Raosoft^1^ as the sampling calculation tool, set at the margin of error of 5%, a confidence level of 95%, and a population size of 350 licensed preschool teachers, the 182 teacher-respondents were selected.

All preschool instructors are women who work in the following Romanian counties: Alba, Arad, Bihor, Bistrita-Nasaud, Brasov, Caras-Severin, Cluj-Napoca, Dolj, Gorj, Hunedoara, Mehedinti, Salaj, Satu Mare, Sibiu, and Timis. We received replies from teachers with age ranging from 23 to 62 years, with a mean age of 42 years. Regarding our respondents’ professional experience in preschool education, they ranged from 2 to 43 years, with an average mean of 20 years. All of our responses hold a government-accredited title and a bachelor’s diploma or higher in the field of preschool and elementary education. As to the ethical consideration of the study, the authors followed the following: (1) permission from authorities to conduct the study was sought; (2) informed consent of the participants was sought before participating in the study; (3) the approval of the Institutional Review Board (IRB) was sought before and after conducting the study.

### Measures

Technological acceptance in ECEC envisages the preschool teacher as a change agent and further presents an extension of the classical TAM, paying particular attention to the specificities of ECEC. The use of the TAM scale in this study provide empirical evidence on the technological literacy of preschool teaches in the Romanian context.

The TAM in ECEC (TAM ECEC) is a tool that assesses ECEC professionals’ technology acceptability in an online learning environment, based on the theory of planned behavior.

The online questionnaire consists of 27 items, rated by respondents on a Likert scale ranging from 1 to 5 (1—strong disagreement, 5—strong agreement). We use reverse scoring for items Q22, Q23, Q24, Q25, and Q27, which means 1 is recorded with 5 and vice versa (Rad et al., 2022).

Eight dimensions ratings are utilized to interpret the data:

• D1. Perceived usefulness,
• D2. Perceived ease of use,
• D3. Perceived enjoyment,
• D4. Intention to use,
• D5. Actual use,
• D6. Compatibility,
• D7. Attitude,
• D8. Self-efficacy.

Higher ratings imply higher level of embrace of technology in the class. In the present research sample, Cronbach’s alpha of the dimensions for D1, D2, D3, D6, D7, and D8 were 0.91, 0.83, 0.91, 0.91, 0.95, and 0.83, respectively, except for D4 and D5 that are represented by single items, reflecting the behavioral intent to use and actual use of technology. The online questionnaire TAM ECEC demonstrated good reliability coefficients with a calculated Cronbach’s alpha of 93%. Regarding each dimension’s statistics, perceived usefulness reported a mean of 2.56 and a SD of 1.12, perceived ease of use reported a mean of 3.07 and a SD of 0.91, perceived enjoyment reported a mean of 3.25 and a SD of 1.03, intention to use reported a mean of 3.17 and a SD of 1.17, actual use reported a mean of 2.92 and a SD of 1.15, compatibility reported a mean of 3 and a SD of 1.04, attitude reported a mean of 2.91 and a SD of 1.11, and self-efficacy reported a mean of 3.87 and a SD of 0.86.

As for the scale overall statistics, results showed a mean of 24.77, with a variance of 48.68, and a SD of 6.97, Cochran’s *Q* of 322.04 at a *p* < 0.01, and a Hotelling’s *T*-squared *F* of 44.29 at a *p* < 0.01 All the above mentioned coefficients bring argumentation in the favor of the validity of the TAM scale.

### Procedures

Technological acceptance in ECEC sees the preschool teacher as a change agent, as well as an extension of the traditional TAM that takes into account the unique characteristics of ECEC. The TAM scale will be used in this study to give empirical evidence on preschool teachers’ technology literacy in the Romanian context. The TAM has been used in several research to determine technological acceptance and forecast how new information resources will be used. Confidence in the use of technology can lead to increased personal control, flexibility, and competent information use, according to the study. First, we translated the online questionnaire into Romanian and amended the original items to reflect the usage of educational technology by preschool instructors during the COVID-19 period. Second, a focus group with 10 specialists from Arad’s Aurel Vlaicu University was convened to obtain feedback. The online questionnaire was modified and changed based on these first results. According to the statistics, e-questionnaire replies take 11–17 min to complete. Finally, a method of convenience sampling was adopted. Preschool teachers were selected at random from a Romanian National Training Program for Preschool Teachers. After learning about the research aims, the instructor consented to participate willingly. After collecting responses, we have created the database in SPSS V.26. For the purpose of predicting preschool teachers’ actual technology usage with NNs modeling, we have used the RBF facility in SPSS.

## Results

Radial basis function neural network is made up of three layers: an input layer, a hidden layer, and an output layer. We employed a standard multiple-input, single-output RBFNN in this investigation. Out of a total of 182 instances, 128 cases were allocated the training sample, accounting for 70.3%, and 54 cases were assigned the testing sample, accounting for 29.7%. In the network information output, the input layer has seven independent variables as covariates: perceived usefulness, perceived ease of use, perceived enjoyment, intention to use, compatibility, attitude, and self-efficacy, and we have chosen the standardized rescaling method for covariates. The hidden layer was designed with five units and Softmax as a activation function. Our RBF was designed with five units, because this criterion yields the smallest error in the testing data. The output layer is represented by the dependent variable, D5. Actual use, being designed with 5 units or data points, as responses to our online questionnaire were collected on a 1 to 5 Likert scale, and using “identity” activation function.

**FIGURE 1:**
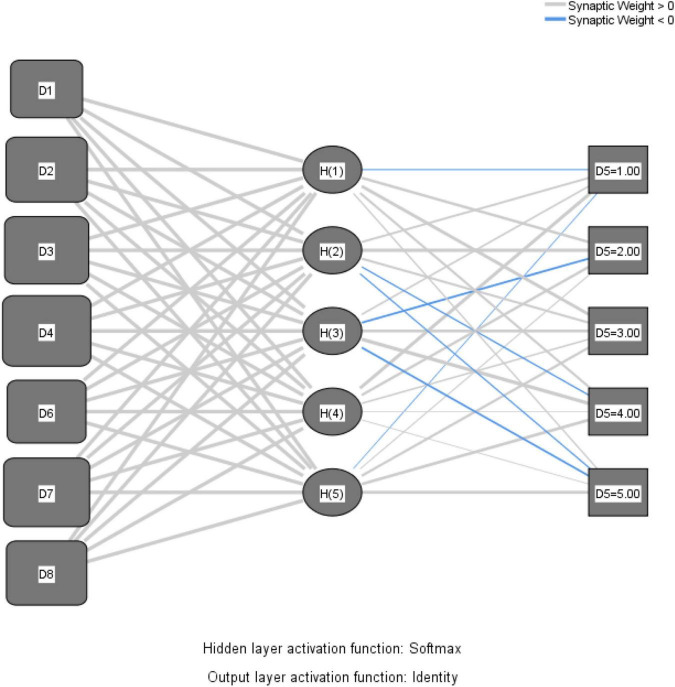
The architecture of the radial basis function neural network.

In Figure 1, the RBFNN architecture is depicted, with all the above-described layers.

In the model summary, we obtained a sum of squares error of 37.5 and a percent of incorrect predictions of 43.3% in the training sample. In the testing sample, we obtained a sum of squares error of 14.88 and a percent of incorrect predictions of 37%. Thus, we can conclude that 63% of the classified data are correctly assigned to the dependent variable, D5. Actual use value, which is a significant rate of correct predictions in the testing sample.

**FIGURE 2:**
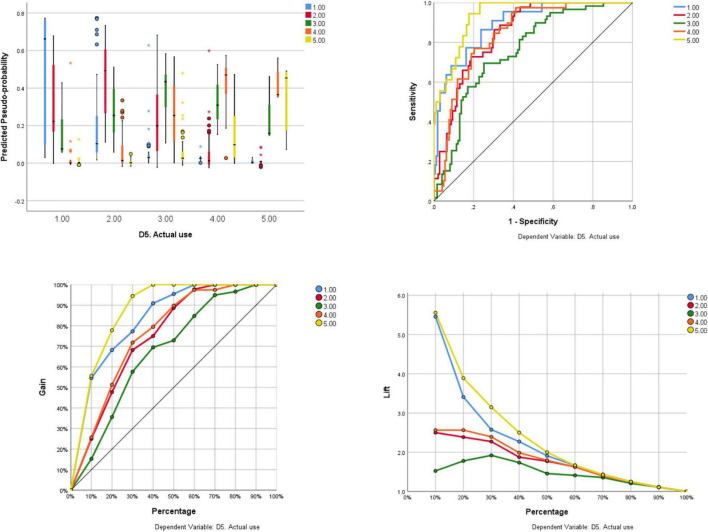
The predicted pseudo-probability, specificity–sensitivity, the gain curve, and the lift curve generated by the RBF algorithm.

Further, in Table 1, we present the correct percent of classification for each data point in both training and testing samples. The lowest correct percent in the training sample was obtained for data point 5, namely 45.5%, with an overall correct percent of 54.7%. In the testing sample, the lowest correct percent was obtained for data point 2, namely 41.7%, with an overall correct percent of 63%. The predicted pseudo-probability, sensitivity-specificity, cumulative gain, and lift chart outputs are presented in Figure 2, evidencing our RDB classification model performance.

**TABLE 1:** Correct classification percentages.

Classification							

Sample	Observed	Predicted					
		1.00	2.00	3.00	4.00	5.00	Percent correct
Training	1.00	9	5	1	1	0	56.3%
	2.00	2	22	8	0	0	68.8%
	3.00	1	9	18	10	1	46.2%
	4.00	0	1	9	16	4	53.3%
	5.00	0	0	1	5	5	45.5%
	Overall percent	9.4%	28.9%	28.9%	25.0%	7.8%	54.7%
Testing	1.00	3	3	0	0	0	50.0%
	2.00	5	5	2	0	0	41.7%
	3.00	0	2	14	4	0	70.0%
	4.00	0	0	0	7	2	77.8%
	5.00	0	0	1	1	5	71.4%
	Overall percent	14.8%	18.5%	31.5%	22.2%	13.0%	63.0%

*Dependent variable: D5. Actual use.*

In describing our model’s performance, we also refer to the gain/lift analysis, which assists in determining the best predictive model among numerous competing models. Gain and lift charts are used to assess classification model performance. They assess how much better one may anticipate to do with a predictive model than without one. The slope presented in Figure 2 steadily lowers as there are fewer records that are actually the class of interest to add, and the model has less potential to give an advantage, as seen in the lift chart. Our RBF performs excellently, yielding a significant “lift” for a relatively small fraction of the ranked data. The area under the curve (AUC) (Table 2) value identify D5. Actual use, when using the RBF model is showing accurate overall performance.

**TABLE 2:** Area under the curve (AUC).

		Area
D5. Actual use	1.00	0.892
	2.00	0.847
	3.00	0.758
	4.00	0.840
	5.00	0.936

An AUC of 0.5 indicates no discrimination, 0.7–0.8 is good, 0.8–0.9 is great, and more than 0.9 is exceptional (Mandrekar, 2010). As we can see in Table 3, for data point 1, the AUC is 0.89; for data point 2, the AUC is 0.84; for data point 3 the AUC is 0.75; for data point 4 the AUC is 0.84; and for data point 5 the AUC is 0.93.

**TABLE 3:** Independent variable importance.

	Importance	Normalized importance (%)
D1. Perceived usefulness	0.117	72.3
D2. Perceived ease of use	0.142	87.9
D3. Perceived enjoyment	0.150	93.0
D4. Intention to use	0.161	100.0
D6. Compatibility	0.135	83.7
D7. Attitude	0.155	96.1
D8. Self-efficacy	0.140	86.6

**FIGURE 3:**
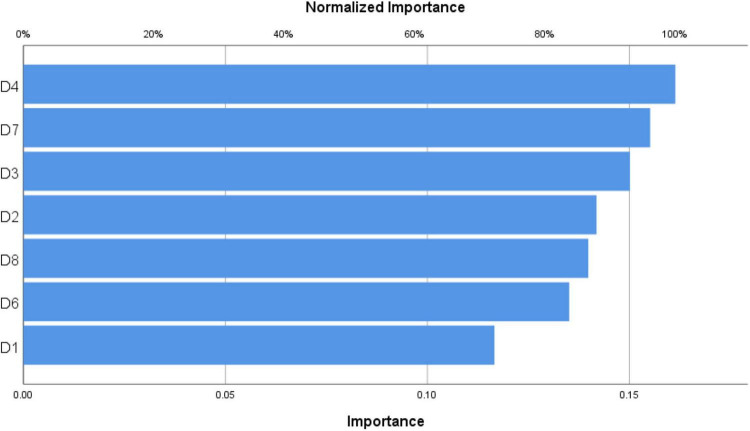
Independent variable importance.

In the training phase of RBF model, results revealed that D1, D2, D3, D4, D6, D7, and D8 were all important factors among all independent variables for D5 prediction, the normalized importance for them were 72.3, 87.9, 93, 100, 83.7, 96.1, and 86.6, respectively (see Table 3 and Figure 3). The most important dimension is D4. Intention to use, which is supported by the theory of planned behavior, which states that behavioral achievement depends mainly on intention.

## Discussion

Although several earlier research have explored and characterized determinants of technology use behavioral intention, not many researchers are focusing on antecedent factors of technology acceptability of preschool instructors, except for the study of Hong et al. (2021). Building and testing a model with numerous impacting elements leads to a more thorough knowledge of the preschool teacher’s technology acceptance. The current study used the modified TAM to explain preschool teachers’ adoption of educational technology during the COVID-19 pandemic, which was motivated by earlier literature. Our adapted TAM took into consideration the following dimensions: perceived usefulness, perceived ease of use, perceived enjoyment, intention to use, actual use, compatibility, attitude, and self-efficacy.

The study employed the TAMECEC as a tool that assesses ECEC professionals’ technology acceptability in an online learning environment. This study included 182 preschool teachers who were chosen at random from a project-based national preschool teacher training program called Inclusive and Qualitative Early Education, which began in Romania in the spring of 2021 during the COVID-19 pandemic.

The present study is an investigation using the RBFNN as an approach to predict preschool teachers’ technology acceptance behavior. We have further employed RBFNN modeling to predict actual usage of technology in class (D5) based on the seven dimensions: D1, D2, D3, D4, D6, D7, and D8. In the model summary, we obtained in the training sample a sum of squares error of 37.5 and a percent of incorrect predictions of 43.3%. In the testing sample, we obtained a sum of squares error of 14.88 and a percent of incorrect predictions of 37%. Thus, we conclude that 63% of classified data are correctly assigned to depended variable D5. Actual use value, which is a significant rate of correct predictions in the testing sample.

This high significant percentage of correct classification represents an important result, mainly because this is one of the first study that applies NN prediction on psychological data, opening up a new interdisciplinary field of research.

In this study, the sensitivity, specificity, and accuracy of the RBF model were all significant. Moreover, the AUC value for recognizing D5. Actual behavior using the RBF model was optimal. Furthermore, predicted pseudo-probability curve, gain curve, and lift curve were in a reasonable performance, which demonstrates an accurate prediction of D5. Actual behavior of embracing technology in class, using RBFNN by testing the dynamical relationship between variables instead of using the classical multiple linear regression analysis, which implies linear associations between dimensions.

Furthermore, for the accurate prediction of D5. Actual behavior of embracing technology in class, using RBFNN by testing the dynamical relationship between variables instead of using the classical multiple linear regression analysis, which implies linear associations between dimensions, proves that the NN approach in predicting an actual complex and dynamic behavior like technology use in ECEC is a robust data processing approach.

## Limitations

In terms of benefits and drawbacks, the RBF network is an artificial NN that employs the RBF as a activation function. The network’s output is a linear combination of the inputs’ RBF s and neuron parameters. In this study, we used RBF networks to classify preschool instructors’ intentions to use technology in class.

Although the RBF has been shown to be an efficient approach for nonlinear process modeling due to its strong function approximation potential, in many cases, the sample set is restricted and the model evaluation error is fixed, making it very difficult to design an optimal network structure for ensuring the generalization capacity of the defined nonlinear process model.

## Conclusion

The use of digital technology in the classroom can help to close the digital literacy gap and prepare children for the future society. There is a need to successfully integrate digital technology in the educational system starting with ECEC in Romania. This study was carried out to identify the critical factors in technology acceptance in ECEC in Romania. The analysis of the results using a RBFNN approach showed the following dimensions: D1. Perceived usefulness, D2. Perceived ease of use, D3. Perceived enjoyment, D4. Intention to use, D6. Compatibility, D7. Attitude, and D8. Self-efficacy, which are significant predictors of D5. Actual use, the preschool teachers’ actual use of technology in classroom, with an accurate prediction rate of 63%.

Our research looked at the modified TAM model in data from preschool instructors in the context of the COVID-19 outbreak. Our study adds to the literature by concentrating on the behavioral intentions of Romanian preschool instructors who were recently involved in online education activities. Our study contributes to the discovery of possible characteristics that influence preschool instructors’ willingness to use educational technology in Romania. If similar research is further applied in other European countries, the conclusions can be used to establish focused practical methods to increase the technological acceptability of preschool instructors in Europe and thus further adopt the objectives of the Sustainable Development Goal (4) Quality Education Agenda for 2030.

This study has an important novelty representing the first approach to use RBFNN in the field of psychology, indicating the good predictive capabilities of RBFNN.

## Data Availability Statement

The raw data supporting the conclusions of this article will be made available by the authors, without undue reservation.

## Ethics Statement

Ethical review and approval was not required for the study on human participants in accordance with the local legislation and institutional requirements. The patients/participants provided their written informed consent to participate in this study.

## Author Contributions

All authors listed have equally made a substantial, direct, and intellectual contribution to the work, and approved it for publication.

## Conflict of Interest

The authors declare that the research was conducted in the absence of any commercial or financial relationships that could be construed as a potential conflict of interest.

## Publisher’s Note

All claims expressed in this article are solely those of the authors and do not necessarily represent those of their affiliated organizations, or those of the publisher, the editors and the reviewers. Any product that may be evaluated in this article, or claim that may be made by its manufacturer, is not guaranteed or endorsed by the publisher.

## Footnotes

1
http://www.raosoft.com/samplesize.html
